# Sustained service: a community-driven framework for longitudinal service-learning

**DOI:** 10.15694/mep.2018.0000112.1

**Published:** 2018-06-05

**Authors:** Don Robison, Alexandra Leader, Maryanne Gathambo, Erin Madison, Alicia St. Thomas

**Affiliations:** 1 Eastern Virginia Medical School

**Keywords:** service-learning, sustained service, social determinants of health, fair-trade learning, capstone

## Abstract

This article was migrated. The article was marked as recommended.

The sustained service four-year longitudinal framework for medical school service-learning is introduced and defined. Framework benefits include: students can engage deeply over time with both the people they serve and their colleagues, they are immersed in the social determinants of health in authentic contexts, and they grow in the expertise required to perform their service over time. The approach starts with a sophisticated community needs assessment that relies on systematic inclusion of community voices, community leader inputs, and systematic and data-saturated inputs. This needs assessment should result in a six to eight item list of the community’s priority needs. All student service is then focused on the primary needs identified in the assessment. Goals of the framework are described: to make a difference with the community’s priority needs; to grow the identity, skills and paradigms required of a community-responsive physician; and to strengthen student credentials through objective qualifications related to their sustained service. The culminating activity is a Capstone research project that focuses on the served population and gives students an opportunity to synthesize their experience. Initial results describing the community impact of service, the focus of service hours, and candid student reactions to the approach are presented. Discussions of findings and conclusions are offered.

## Introduction

Most disease is the result of the social conditions in which people live ( Goldberg, Baxley, & Fancher, 2017). Socioeconomic, behavioral, or environmental factors account for about 80% of health outcomes while medical care accounts for 20% of outcomes ( Berman, Durning, Fischer, Huwendiek, & Triola, 2016). The larger part of undergraduate medical curricula typically focuses on this 20% of health outcomes and the other 80% of health outcomes-the socio-ecologic determinants of health (SDOH)--are addressed inconsistently or inadequately. Service-learning is an ideal medium for learning about and experiencing SDOH, and has an established track record in this regard. But, service-learning experiences are often brief, informal, or inspired by student or faculty values rather than voiced community needs.

This article provides an overview of a framework for a four-year longitudinal service-learning approach for undergraduate medical education that starts with a rigorous identification of the community’s priority needs, a tailored program structured around those identified needs, unique experiential designs, complementary specialty-based qualifications and eLearning, a strong support infrastructure, and a synthesizing Capstone experience focused on the served population. This is a community-driven approach in that fair trade learning practices ( Hartman, 2015) are employed in needs identification, initiative selection, execution, evaluation, and improvement processes. Fair trade learning is defined as “.. a global educational partnership exchange that prioritizes reciprocity in relationships through cooperative, cross-cultural participation in learning, service, and civil society efforts” ( Campus Compact, 2018).

Service-learning is not new in medical education and has been defined various ways. Seifer, Hermanns, and Lewis (2000) defined it as “.. a structured learning experience that combines community service with explicit learning objectives, preparation, and reflection. Students engaged in service-learning are expected not only to provide direct community service but also to learn about the context in which the service is provided, the connection between the service and their academic coursework, and their roles as citizens” (p. 9).

Seifer et al. (2000) described key ways that service-learning differs from traditional clinical education: Service-learning presents a unique balance between service and learning objectives. It emphasizes reciprocal learning, reflection, and the integral role of community partners.

The sustained service framework introduced here shares these characteristics common to effective service-learning, but also emphasizes:

• Student service that ideally addresses carefully identified priority community needs
• Deep student engagement over time in service and personal relationship, and
• Strengthening students’ professional credentials through service-related qualifications.

## The sustained service framework

The sustained service framework employs a four-year longitudinal service pattern that allows students to deeply experience and learn the things relevant to their served population and service activities. The approach includes unique community interactions, a singular mentoring process, and a robust tracking and support infrastructure. This article introduces the distinctive goals, strategies, processes and tools employed in the sustained service approach. The first section provides an overview of the community-driven needs identification process used in sustained service, the second outlines how a program can be structured around the community needs using service pathways and tailored service initiatives, the third section describes the key operational elements of the approach. Data depicting student service patterns, service outcomes, and student perceptions are offered.

**If wildly successful..** If successful, this approach to service-learning will result in two ultimate outcomes: first, over time, there would be discernable improvement in the community needs targeted by the service; and second, graduates will demonstrate the targeted paradigms, values, and skills through their future choices to engage in their communities to make a sustained difference.

## The community needs to student service

### Community-Driven and Data Supported: Identifying the Community’s Priority Needs

**Figure 1.**
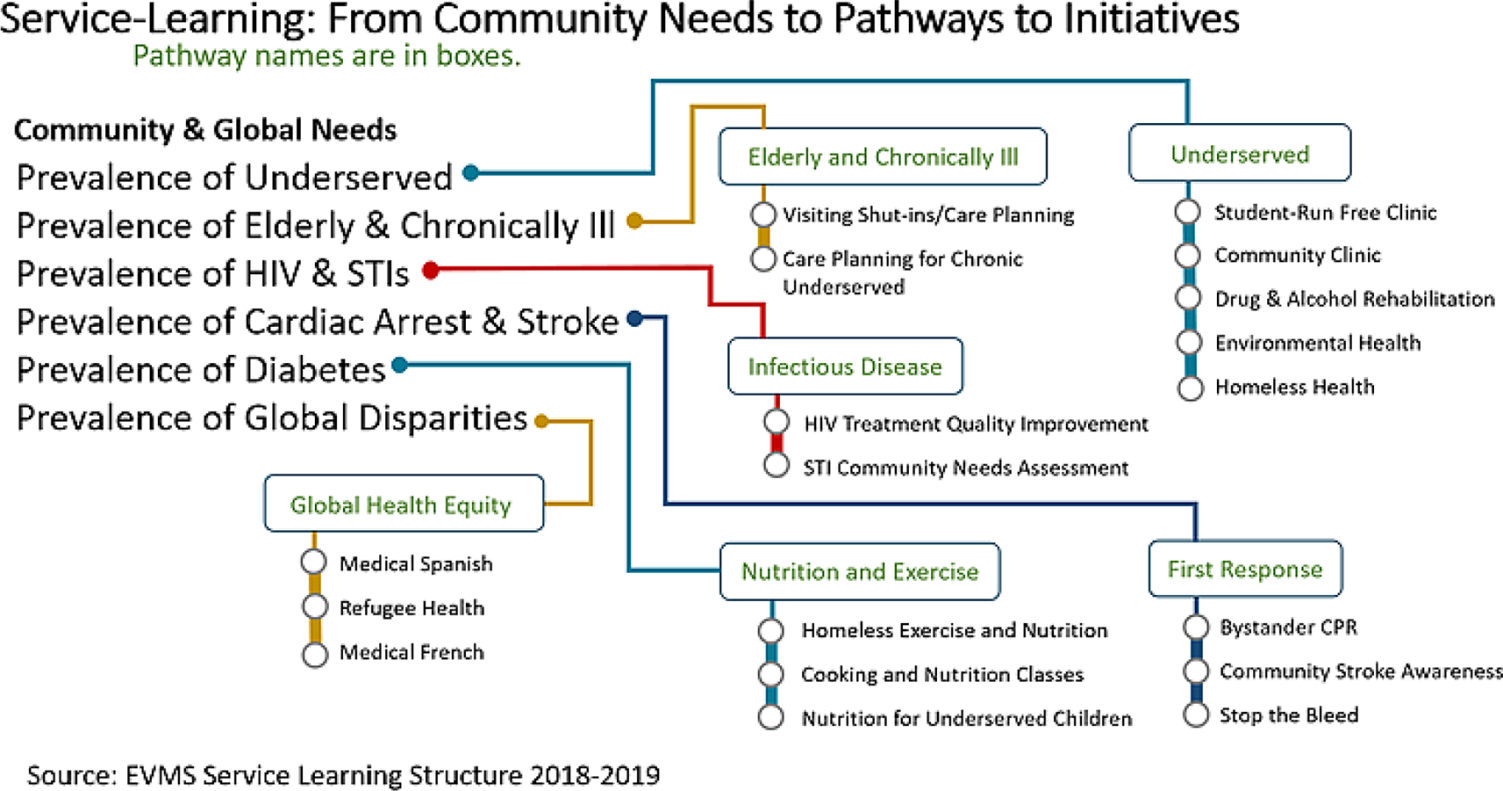
Relating needs to service pathways and then to individual initiatives.

An integral part of the sustained service framework is that the service efforts of students address meaningful community challenges. To accomplish this, all service is aimed at priority community needs. The approach begins with a methodical assessment process to define six to eight of the highest priority needs of the community. Service pathways are then created to address each priority need. For example, in the Norfolk, Virginia area, Diabetes Mellitus Type II is prevalent. The need to prevent diabetes was expressed by local community members and leaders and validated from multiple data sources (Community Health Assessments and Robert Woods Johnson data). The service pathway “Nutrition and Exercise” was then created to focus service teams towards addressing the need. Next, with community, student and faculty representatives, specific initiatives were planned that impact key populations or issues related to the need. For example, in partnership with a local fitness center, students work with “Project LIFT” to help run a fitness, nutrition, and job skills effort for homeless individuals. Another service-learning group works in public housing projects conducting nutrition and cooking classes. In this way, the individual initiative addresses the priority community need through the pathway associated with that need. Figure 1 presents the progression from need to pathway to individual initiative for the current service-learning initiatives at Eastern Virginia Medical School (EVMS).

**Figure 2.**
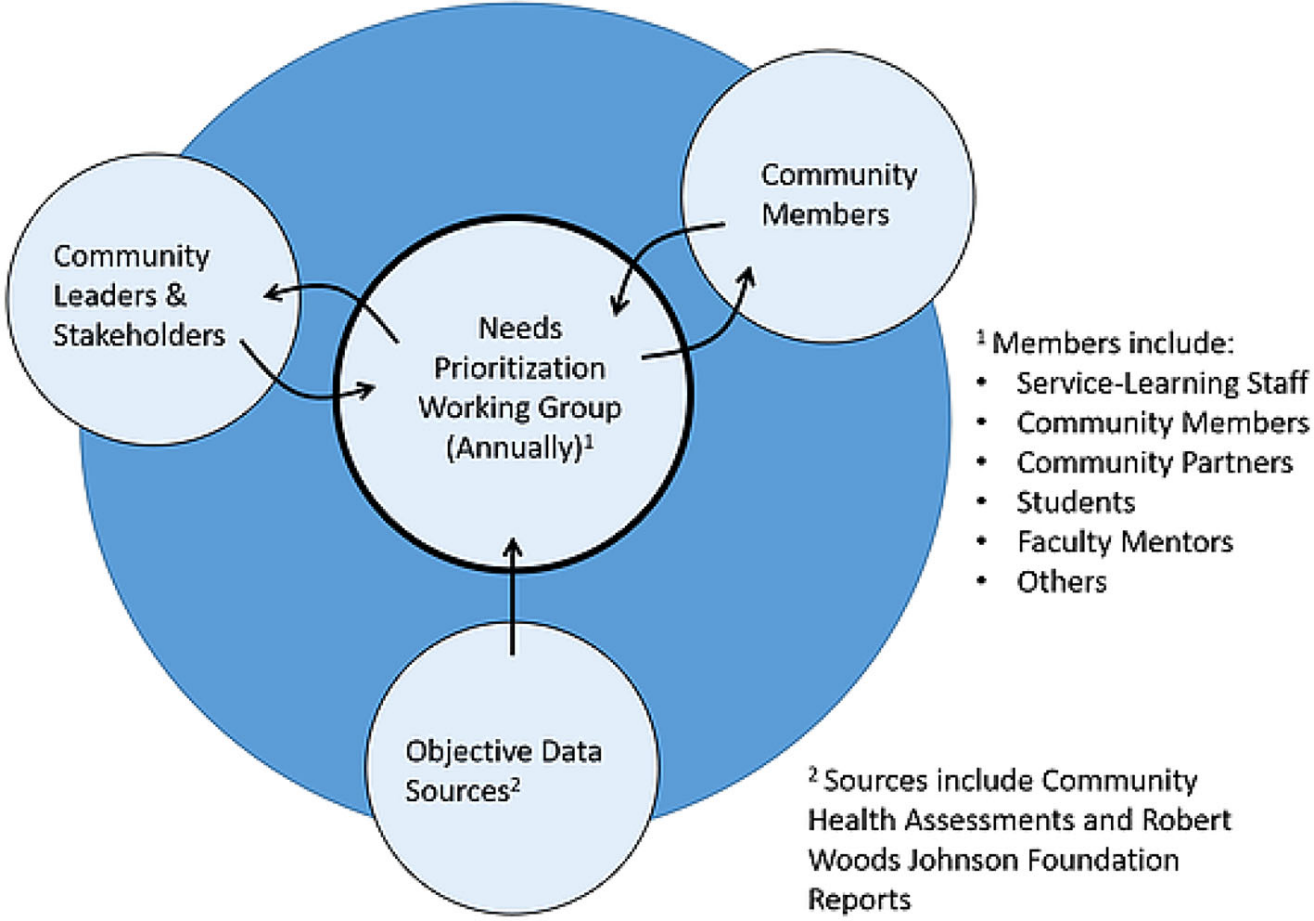
The three key sources of community priority needs information. The voices of community leaders and members are evaluated along with pertinent data. Final consensus is the result of clarifying conversations. The end goal is a consensus priority list that aligns with data.

**The process for identifying the community’s priority needs.** The goal in identifying the community’s priority needs is to produce a list of the top six to eight needs, and then focus all of the service-learning activity that follows on those needs. Three discrete sources of information form the foundation for the priority needs identification process (See Figure 2). They are: the voice of community leaders and stakeholders, the voice of community members (with care to include those typically without a voice), and objective data sources and local community health assessment (CHA) reports. The voices of community members are sought before data-based sources of information because this is the most fragile source of information. In the sustained service framework, community voices are given first priority and detailed data and systematically developed reports are subsequently used to validate or disambiguate direction. The process is iterative, particularly in interacting with community members; it is an ongoing relationship and continuing conversation that must be nurtured.

As practiced at EVMS, the voice of community leaders regarding priority needs is straight-forward. The ‘Healthy Hampton Roads Coalition’ publishes a prioritized needs listing annually. Of course, it is important to follow up and ensure consensus, but this source is readily available. Hearing the voice of community members is more challenging, and for this purpose Community Advisory Boards (CABs) are convened. Generally created for broader purposes, the CABs allow for ongoing conversations about the community’s needs. These are not one-and-done interactions, but require initial discussions, consensus, and ongoing clarification and alignment. Care is taken to develop trust. The third source of information is comprised of multiple sources of epidemiologic data or systematic reports: in the U.S. this includes data sources such as Robert Woods Johnson Foundation data, Macy Foundation data, and local hospital and health department CHAs. All this information is then collated and reviewed by a community-based working group that includes students, faculty mentors, community representatives, community partners, program representatives, service-learning representatives, educational specialists, and service partners. This working group studies the input information from the three sources described, and identifies the priority needs. The consensus product of these deliberations is a *Priority Community Needs Listing* that describes six to eight needs.

Because this is not just service, but also learning, it is important to filter the needs listing through the lens of student learning objectives. It is difficult to imagine, however, a priority community health need that could not be relevantly addressed through service-learning.

**Structuring service towards priority community needs: service pathways and student initiatives.** Once the community’s priority needs have been identified, the next step is to develop service pathways that focus student service on those needs. Based on Eastern Virginia’s needs, five unique service pathways were developed to address local needs. An additional pathway, Global Health Equity, was developed to specifically address those local needs that represent global health inequities.

***The process for identifying service pathways.*** The community’s priority needs should be described in terms of the current state using statistical descriptors if possible (e.g., “21.3% of Norfolk children live below the poverty line”). The associated service pathways that address the needs are named in terms of either the served population or a key solution. Practically speaking, the service pathways must be relatively stable because several enduring program components are developed for them. With each service pathway, tailored certification processes are defined. For example, for the *Nutrition and Exercise* service pathway, there is an associated eLearning course that gives students a depth of expertise in nutrition-oriented medicine and results in an institutional Nutrition certification.

When the Needs Prioritization Working Group identifies a new service pathway, it must develop and document a transition plan to bring that new pathway into the program. As a value, when a new service pathway is added, an old one should be gradually eliminated. Such a transition can take four years since students are already assigned to four year initiatives within each pathway.

**Table:**
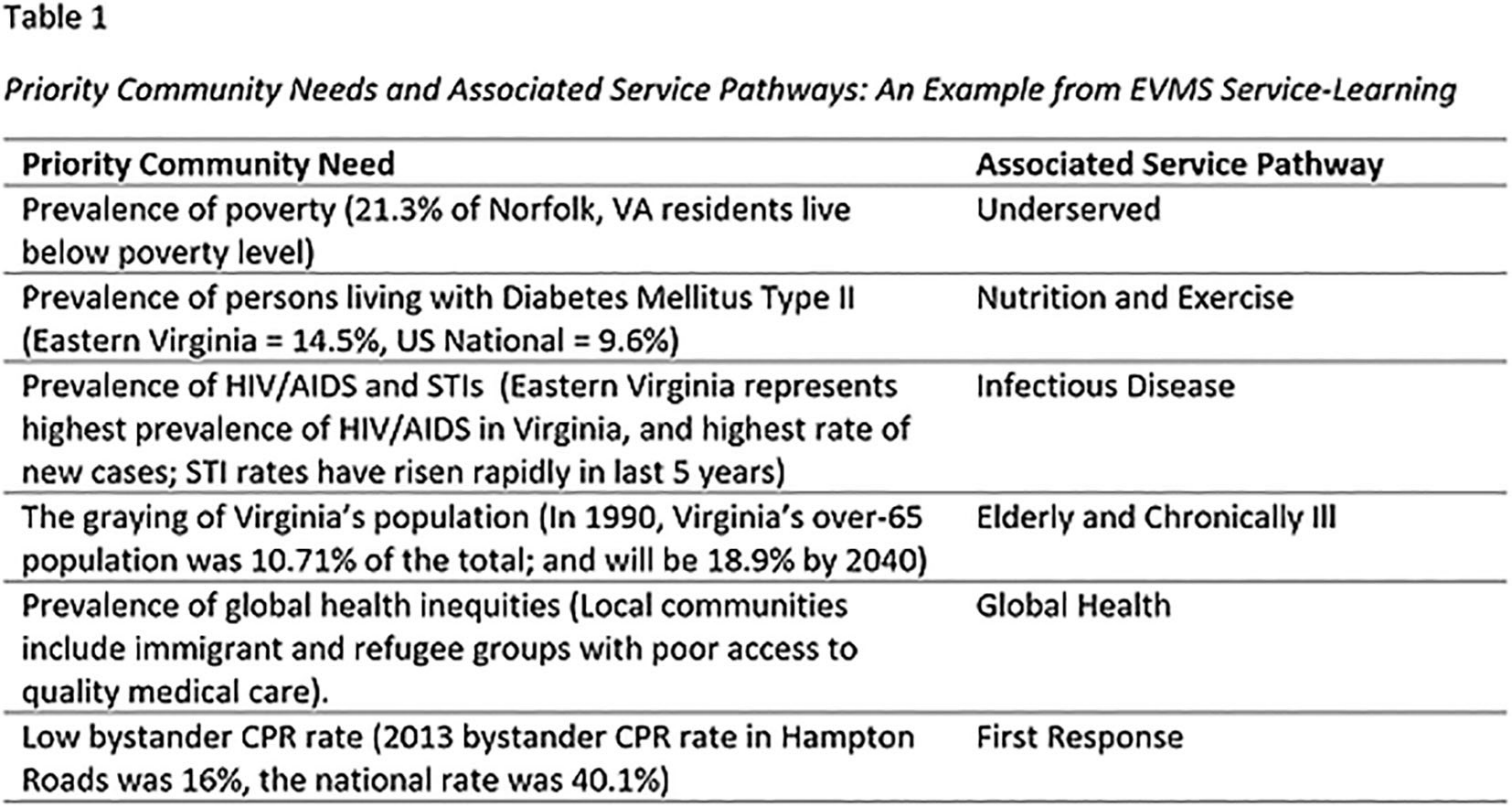


***The process for developing individual initiatives within the service pathways.*** This task is similar to the challenge any service-learning program faces. However, in this case, only initiatives that directly address identified priority needs through service pathways are considered for incorporation into the sustained service framework. There are limitations to this approach: it is possible that initiatives that might be attractive to students, or may encourage productive community partnerships, may be precluded. Experience has shown, however, that by identifying several priority community needs, there are a wide variety of excellent service opportunities available to students.

*Characteristics of successful longitudinal initiatives:* The best initiatives have a practical task of moderate difficulty that can be feasibly implemented, but also relate to either a population or health phenomenon that is associated with complex variables. In other words, the successful initiatives have a concrete action students can perform instantly, and a researchable challenge. That said, initiatives can vary widely in type. Some initiatives are research oriented, some very hands-on, some relational, and others are more task-oriented. Because of the importance of modeling in this context, the most successful service-learning initiatives have strong faculty or community mentors.

### The Student Pattern of Service

Students generally serve four years in the same service project. Consequently, great care must be taken so that (a) students have a clear idea of the requirements of the initiative, (b) the available initiatives are worthy of sustained service, (c) there are reasonable opportunities to change initiatives if necessary or desired, (d) easy-to-use service reporting methods are employed, and (e) any student certification processes are built into the student routine in ways that are not overly burdensome.

**Student service initiative selection.** The mission and activities of each service initiative should be carefully described in multiple communication streams. At EVMS, a service-learning catalog is published every year that describes each initiative, its purpose, and associated service pattern. This content is also provided in the school’s website so that prospective students may access it. In addition, during the first week of classes, a Service-Learning Fair is conducted. Tables for each initiative are staffed by second year medical students who answer the incoming students’ questions and create informational posters for the various initiatives. A soundtrack, popcorn, handouts, and prizes create a festive atmosphere. Initiatives requiring foreign language competency (Medical Spanish and Medical French) offer written and verbal assessments required of students interested in participating in these initiatives. Students then have 72 hours to submit their top three choices for initiatives in a phone app. Students are randomly sorted and then assigned to initiatives based on their top choices. In this way over 95% of students have received their first or second choice of assignments. At EVMS, the overwhelming majority of students reported they would have chosen their previously assigned initiative if they had to select again.

**The importance of providing an opportunity for change.** Because this is a sustained service framework, it is essential that students value their service initiative. For various reasons, this is not always the case. It is important that an opportunity to change initiatives be provided. At EVMS students are allowed to transfer between initiatives for the first two months of the first year and the first two months of the second year of medical school. In practice, it is advisable that some process exist to facilitate other in-year transfers. The essential point is that if the expectation is that students commit to one service initiative for four years, there must be a way they can escape unpleasant, fruitless, or personally irrelevant service assignments. In the EVMS context only 2% of students have requested transfers.

**The rhythm of service.** Most students gravitate to meaningful service. But, since this is medical school, and student time is limited, no students maintain commitment to what may be perceived as a waste of time. Therefore, after initiative assignment, the most important first steps are (a) providing opportunities for social connection with other members of the team, (b) having students meet persons whom they will serve, (c) giving the students an orientation to the skills related to their particular service initiative, (d) getting a good start in service, and (e) clarifying expectations for the service pattern.

In an effort to strike a balance between students serving enough to have personal and community impact, but not making service so burdensome that it distracts from their studies or becomes an extrinsic motivator of its own, the required amount of service is 15 hours per year. As practiced at EVMS, students may work with their initiatives more than 15 hours, but that extra time is clearly identified as volunteer time. The actual rhythm of service for those 15 hours varies wildly between initiatives. Some initiatives deliver a service (e.g., training) and stick to rigorous time schedules, others are more relational, and still others focus primarily on community research.

**Reporting service.** In any service context-but especially in a sustained service context-it is essential that there be an easy-to-use reporting system for student activity. A phone app, or accessible web site, is recommended. At EVMS, a relatively simple phone app reporting system acts as the students’ reporting connection, enabling students to report and allowing leaders to monitor their service. Students also report where they conducted their service from either a drop-down menu of common sites, or manual entry of an address, and the system determines student eligibility for mileage reimbursement and emails the student a mileage claim if the student qualifies. This feature adds value to use of the system from the students’ perspective. Data captured through the reporting app is then imported into a more complex database so that student service may be monitored and analyzed. Commercially available app and data-management systems can provide this capability, and there are widely-used service reporting systems available.

**eLearning addresses leadership, followership, systems and SDOH objectives.** Several performance objectives are addressed through hybrid courses using eLearning and/or optional classroom sessions. These objectives are drawn from performance goals in the leadership, followership, systems thinking, and SDOH domains. Students may choose one of two methods for completing these requirements: they may be satisfied through completing seven online self-paced tutorials, or, students may complete the requirements through optional class sessions. Six lunchtime “Bagel and Leadership” meetings offered during the year at EVMS address the objectives of the eLearning modules. About 40% of the students choose the lunchtime sessions over eLearning. Students may also choose to complete some of the requirements online and the remainder in the optional class sessions.

**Service pathway certifications.** Perhaps the most challenging goal of the sustained service framework is the identification or development of certifications that can meaningfully accompany the service. Several of the specific initiatives have built-in qualifications. For example, in teaching Bystander CPR or Community Stroke Awareness, there are qualification courses that students must take prior to acting as instructors. But, for most initiatives, there are no built-in certifications. Therefore, certifications with identifiable performance criteria have been associated with each service pathway that does not have built-in requirements.

**Figure 3.**
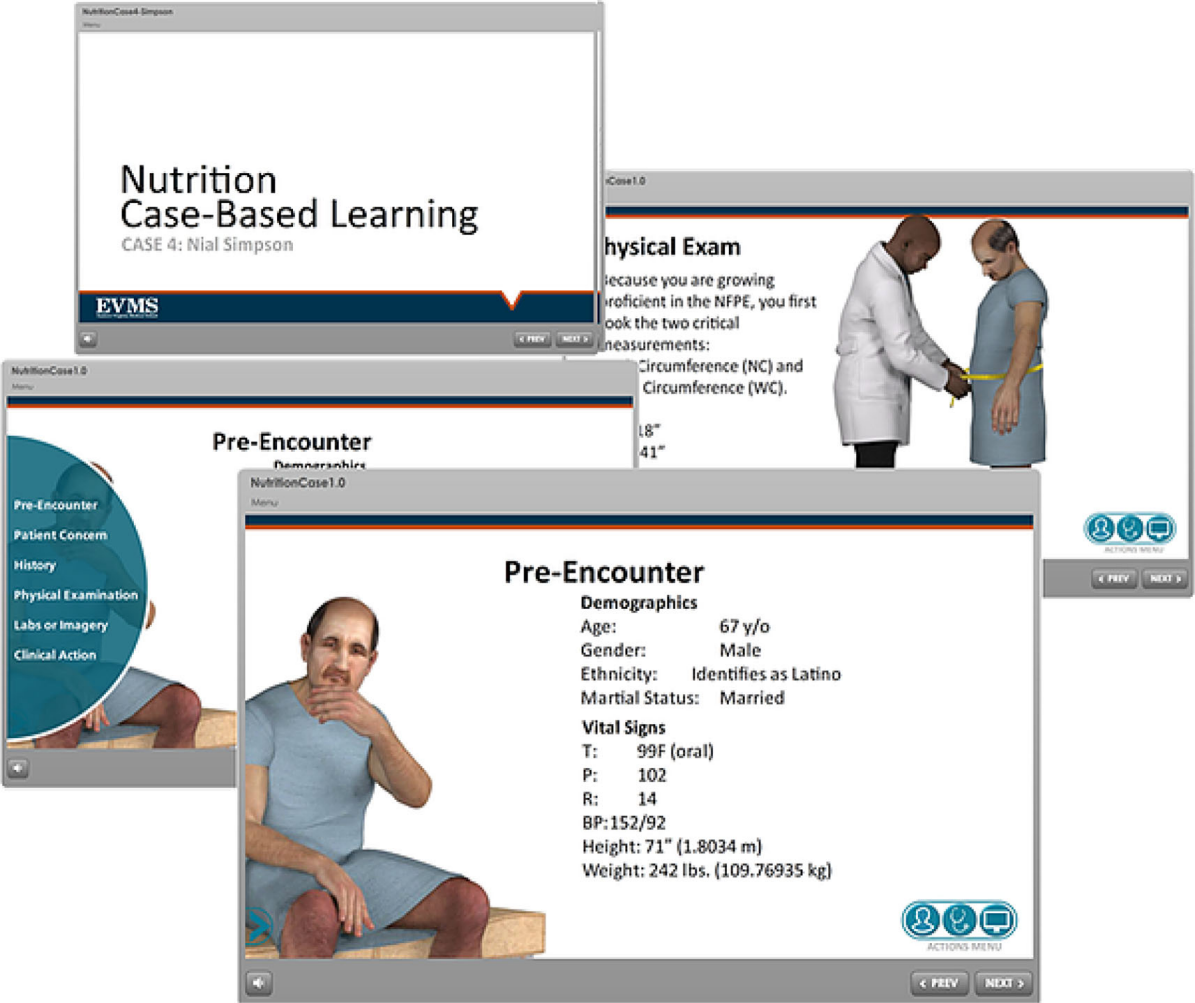
Screenshots from simulated nutrition-focused physical examination. This Nutrition eLearning certification is associated with the Nutrition and Exercise Service Pathway. These pathway-associated certifications add strength to student resumes in a competitive world.

For example, at EVMS an eight-part eLearning tutorial in Nutrition was developed in conjunction with the Nutrition and Exercise Service Pathway on criteria provided by Michael Rothkopf, MD, FACP, FACN. Dr. Rothkopf was serving as the President of the National Board of Physician Nutrition Specialists at the time. Students complete the course, including four online Nutrition Focused Physical Exam interactive cases, then take the Nutrition certification exam. If successful, the students are institutionally certified In Nutrition. In the Nutrition eLearning certification, students interact with virtual family members that are used throughout the MD curriculum ( Robison et al., 2017). Content is carefully coordinated with standard curriculum content to ensure there is minimal duplication. Figure 3 presents screenshots from one interactive case used in the Nutrition Certification.

**Capstone.** The optimal learning experience-whether short or long-follows a profile similar to a story plot line. Parrish (2009) described optimal learning experiences as having “beginnings, middles, and endings” (p. 516) and made the point that ‘endings’ should integrate everything that has happened to that point. Late in the fourth year of undergraduate medical school, students submit Capstone projects that focus on the health challenges faced by their served population. These projects can take on many forms from quality improvement projects to true community health research, but all are aimed at getting students to synthesize the many lessons of their sustained service, and formulating principles for their future practice.

### Key Goals and Strategies of Sustained Service

**Paradigm Building.** In his classic article about confronting the “hidden curriculum,” Hafferty (1998) proposed that medical educators focus on what students learn, not just what they are taught. He persuasively argued that much of what is learned is informal-through relationships-or even “hidden” in the milieu of the medical education experience: in policies, structures, and unintentional social norms and jargon. The result of the whole of the medical education, then, is not just an acquisition of identified learning and performance objectives and skills, but of paradigms (some constructive and some not) about how things “really work” in medicine. These practice-related paradigms direct future choices students make as physicians, and can make a difference in the intensity of a student’s future community responsiveness.

In the sustained service framework, the category of “paradigm” is an intentional taxonomic class (similar to knowledge, skills, and attitudes) and objectives are articulated relating to valued paradigms. As described, a paradigm is how one believes the world really works-whether in a micro sense (e.g., how an effective medical exam should be conducted) or in a macro sense (e.g., beliefs about the origins and maintenance of health). Target paradigms in sustained service include systems thinking and socio-ecologic determinants of health.

**Developing Community-Responsive Skills.** If a student accepts the paradigm that health happens in the context of socio-ecologic determinants, and in community or intimate social networks, then part of the challenge has been met. But, in addition to constructive paradigms, students need specific community-oriented skills to make a difference. Students should acquire skills that help them effectively engage with both community leaders (who most often already have a voice) and community members (many of whom may not have a voice). Students must be able to effectively search locally available health data to develop an objective picture of community health. In addition, to effectively respond to community needs, students must acquire and practice team-building and collaborative skills. The urban planning discipline developed the concept, wicked problem, which describes a complex problem for which there are no black and white right or wrong answers, only better or worse solutions. Examples of wicked problems are ‘world hunger’ or ‘urban blight’ (see Rittel, 1973). The wicked community problems students will encounter as physicians will best be addressed by groups and teams working together. It is critical that students acquire the paradigms and skills to work effectively in these contexts.

**Focusing on Mission.** Service-learning is inherently social in many respects: students connect with people in the community, participate on a team, and must maintain constructive and responsive relationships. As the sustained service framework is exercised, the very act of identifying priority community needs is an ongoing social interaction with community members. It is important to note that early student assessments of EVMS service-learning initiatives have been influenced by perceptions of social connectedness. Long-term, however, it is the importance of the mission of the initiative that drives student evaluations of their service. At EVMS, by the end of the second year of medical school student perceptions of the importance of the mission of their service is significantly correlated with whether or not they would recommend their initiative to new students. On the student evaluation survey (seven Likert-type items on a 5 point scale, “Strongly Agree”=5 and “Strongly Disagree=1), the survey item “I would recommend my service initiative to a new student” significantly correlated with the survey item “The mission of my service initiative is important” (r(93) = .54, p < .01).

**Developing the community-responsive physician identity.** That identity has a powerful energizing and directive influence on behavior is both intuitive and supported by the literature ( Erikson, 1980). Seeing oneself as a “father” or a “mother”, for example, provides direction and motivation towards behaviors consistent with those valued personal identities. Self-perceptions of identity have a profound motivational influence on the direction and intensity of behavior.

***Align service experiences with student aspirations.*** Most students enter medical school wanting to make a difference in their world. The kinds of things they aspire to do and be are almost always the same things medical educators and future patients hope for them. There is a remarkable nexus of aspiration as students enter medical school. Too often, the negative “hidden curriculum” discourages students along the way ( Hafferty, 1998). Intentionally building towards the identity of community-responsive physician is a chief goal of sustained service and an effective counter-point to the hidden curriculum.

Erikson (1980) proposed that the search for identity is one of the chief motivators for people in their teens and early twenties, and it continues to be a key motivator through the rest of life. This search can be thought of as consisting of a quest to find the answers to two broad questions: “Who am I?” and “What are my capabilities?” This first question, “Who am I?” focuses on the unique attributes of the person including things like personal values, paradigms, physical characteristics (e.g., hair color, body type, gender), personality characteristics (e.g., extrovert or introvert, friendly or unfriendly), and general capacities (e.g., intelligent versus unintelligent). The second question, “What are my capabilities?” focuses on aptitudes and talents. This second question is strongly motivating because it helps inform the first question, and it helps direct choice behavior. The two questions are not separate; for example, many people do things simply to give themselves information about their own capabilities.

Identity formation is the process of defining one’s individual self, it involves ‘trying on’ different roles and identities ( Jazvac-Martek, 2009), of finding models that provide an aspirational target, and of interacting with others to find a preferred social space. Identity formation involves reflection and debate, and most of all, time. Service-learning can provide an ideal medium for encouraging the formation of a community-responsive physician professional identity. In the sustained service framework, five main tactics for encouraging this identity are employed: 1) great care is taken in selection of facilitators who will serve as referent role models, 2) service initiatives are pointed at priority community needs, so students are working to make a difference where it counts, 3) opportunities for success in the context of community work are provided, 4) winsome visual references tie initiatives to student aspirations, and 5) ‘missional communities’ are intentionally created and nurtured. So, for example, a student does not merely work on the LIFT program with homeless individuals (a worthy action on its own), but the student is part of a dedicated team of students working to address the health of persons who are homeless, with a dynamic role model. The student is working on a community need that is important. While it is not guaranteed, the goal is that the student will self-identify, saying things like, “I *am* a person dedicated to the underserved, and I will be a physician with that same commitment.”

One example of a winsome input to identity formation could be woven patches (with associated temporary tattoos) created with student assistance for each service pathway or initiative. An incoming student at a Service-Learning Fair can pick up a patch as she considers which service-learning initiative she will represent with her service-learning commitment throughout medical school. These patches can be fun, but also encourage identity in much the same way that a white coat or military insignia might.

***Carefully recruit essential role models.*** Bandura demonstrated that models have a dramatic impact on choice, performance intensity and on appraisals of self-efficacy. Particularly powerful are the actions, attitudes and paradigms demonstrated by a like-respected role model (1977). A like-respected role model is a model who-in many ways-is similar to the student in the student’s perception. When the student looks at a model who in many respects looks and acts like him or her, then the actions and outcomes related to the model are more salient. Therefore, to impact student values and encourage identity formation, it is valuable to select initiative facilitators who are not only experts in the field, but who students can assess as being like them in salient ways. Does this mean initiative facilitators must be young? No, it does not, but a youthful passion for the initiative is a necessary attribute for a facilitator. And, when a facilitator is young and also boasts both expertise and passion, students are likely to “catch” both the enthusiasm and model more readily ( Bandura, 1977; Lockwood, 2006). Careful selection of role models is an intentional element of the sustained service framework.

**Encourage intrinsic motivation and minimize reliance on extrinsic motivation.** There is always a debate about the virtue of accountability and grading. The sustained service framework could be executed under either a graded or pass-fail system, but over-emphasis on extrinsic motivators is discouraged (see Deci & Ryan, 1975). One of the key goals of this framework is long-term energetic community awareness and responsiveness. If extrinsic motivators (excessive course requirements, required hours, or even personal recognition) are too powerful, intrinsic motivation towards the long-term goal will be diminished. Therefore, great care must be taken to balance intentionality with perceived freedom of choice. To this end, required hours are minimal (15 hours per year at EVMS), while allowing for more volunteer hours if the student chooses.

***Use ‘pull’ versus ‘push’ strategies.*** Also, to the degree practical, “pull” strategies are incorporated. For example, giving students an option for how they will fulfill requirements (like the “Bagels and Leadership Lunch” versus eLearning option described earlier) provides students with some choice in their service. Creating fun or novel experiences that coincide with service is another “pull” strategy: they draw students in rather than pushing them through requirement alone. A strategy like providing an option for how learning requirements may be met builds intrinsic motivation two ways: for the students who attend the classes, they know they *chose* to attend, and for those who did not, they know they could have, reducing the long-term negative motivational impact that could be associated with the requirement.

***Encourage the development of missional community.*** Students (all of us, in fact) have a pervasive need to ‘belong’ in community. It may be among humankind’s most fundamental motives ( Baumeister & Leary, 1995). For those who have experienced it, engaging in a valued mission in concert with others of like-mind is a powerful, even identity-forming event. The effects are more pronounced in immersive service (like that experienced in international service-learning), but can be experienced in local contexts.

***Consider “Flex” service opportunities.*** Many students wish to become involved in the other service initiatives, which is difficult since the service required of their primary initiative may be challenging on its own. One solution to this is allowing a certain number of the required service hours to be “flexible” hours. That is, students may serve in another initiative for a specified number of hours annually. In the case of EVMS, each initiative assigns the number of “flex” hours they allow. For some initiatives that have particularly busy schedules, the “flex” hours may not be offered, for others, they may offer up to three a year. This is positive in several ways: a) it encourages joint health fair type efforts, b) it encourages students to try different types of service, and c) it relieves some of the tension that may stem from the sustained longitudinal service commitment. Many students choose to fulfill all required service-learning hours with their primary initiative and further voluntarily engage with other service-learning activities.

**Optimizing the volunteer engine.** Volunteers make sustained service work, and their energy is a model for students if the program is successful.

***Service-learning is not volunteerism.*** The service-learning literature is replete with the assertion that service-learning is not volunteerism ( Seifer et al., 2000), or that it is not *just* volunteerism. And the point is well-made: service-learning is not volunteerism, particularly not in the sense that it is a unidirectional paternalistic activity, as volunteerism is sometimes perceived ( Jacoby, 2015). Nor is it voluntary as exercised in this framework-service is a required and integrated curricular component. However, it is important to realize that the discretionary energy characteristic of dedicated volunteers is exactly the kind of energy encouraged through service-learning.

***Volunteers are critical.*** Service-learning is not voluntary, but volunteerism provides the critical engine for it. The majority of mentors-perhaps the most important component of the sustained service approach-are volunteers. All of the student leaders-also key in this approach-are volunteers. If service-learning is wildly successful long-term, the students’ futures would be characterized by their choices to expend focused energy on behalf of their communities-whether as volunteers or as dedicated community-responsive physicians. Therefore, it is true, service-learning is not volunteerism, but volunteers provide key energy to power it, and the ongoing volunteer spirit is a key measure of success.

### Integration of Global Health Principles and Opportunities

*“If your neighbor’s house is burning, you are not safe if you lock your doors.”* ( Pitt, 2016)

A key part of the EVMS expression of sustained service includes “Global Health Equity” as a service pathway in recognition that pervasive global health inequities have significant ramifications both globally and locally. Global health inequities are, by their nature, local community health needs. In this service pathway, sustainable partnerships and bidirectional capacity-building with different local immigrant and refugee communities are reinforced and informed by relevant global health experience in international settings. The foundational skills, cultural humility, and commitment fostered by the Global Health initiatives in a local context are solidified, challenged, and ultimately strengthened by vital international training and service opportunities. The Global Health Equity Pathway nurtures the development of physicians whose inclusive community-responsiveness is sustained regardless of where their community may be rooted.

There is debate about the value of international service-learning (ISL). Similar to more generalized controversy regarding the ethical nature of short-term global health missions, critics of ISL ask if students temporarily engaging in international communities and cultures can make a positive impact, especially amidst health determinants of extreme poverty and structural violence. They ask questions such as, “Aren’t student international service-learning trips simply a form of mission-tourism” ( Jacoby, 2015)? The concerns are legitimate, as there are countless historical examples of ill-conceived short-term international health efforts that have had detrimental consequences for the local communities. On the other hand, by building sustainable partnerships to facilitate ongoing collaborative international service initiatives, addressing community-identified needs while dignifying the local community, such ISL efforts can embody the very principles upon which the Sustained Service framework is anchored and can even strengthen the local health care systems in these international communities. Ultimately, viewed as a learning and identity-formation vehicle for the service-learner, and carefully incorporating fair trade learning practices (i.e., working closely with partners in-country), ISL can have enormous value in creating global awareness, developing a global health paradigm, and encouraging learner commitment to global health equity.

It is essential to recognize that global health opportunities exist both locally and internationally. In Eastern Virginia, for instance, there are immigrants and refugees from many different countries and people groups, all with unique health challenges in the U.S. The commitment to work for global health equity is not a commitment to work internationally in service-learning; it is a commitment to advocate for communities suffering health inequities wherever they may be.

Perhaps one of the chief values of ISL or any immersive service opportunity is the experience of “missional community.” There is a synergistic motivational phenomenon that occurs when students gather in an immersive environment to work on an issue they believe in. The focus and camaraderie are rare experiences, and the friendships formed in such circumstances can be very strong. In fact, they can be identity-forming experiences. One of the chief returns from having students work in immersive projects is that they get to experience this missional community. The intention is that they would then seek to create or re-live such community in their professional life.

## Results

### Community Impact

The ultimate ends of sustained service are measurable community outcomes and measurable student outcomes. For the 2017-2018 academic year, EVMS operated 15 different initiatives with 15 different outcome goals. Rather than list all of the targeted community outcomes here, we provide one illustrative example.

**Bystander CPR: An Illustrative example.** In 2013 the rate for bystander CPR (citizens offering CPR support) in the U.S. was reported to be 40.1% for out-of-hospital cardiac arrest cases ( American Heart Association, 2018); but in Norfolk, VA, bystander CPR was performed in only 16% of out-of-hospital cardiac arrest cases. In an effort to close the gap, EVMS started a Bystander CPR service-learning initiative in the fall of 2013. The ultimate goal was to raise Bystander CPR rates in the city, but a second goal was to explore root causes for the low rate. Since then, students developed surveys exploring root causes for low Norfolk Bystander CPR rates and have administered them to 712 individuals. Students have also conducted Bystander CPR training for over 700 individuals in the community. Anecdotally, a day after Bystander CPR training session was administered to Norfolk City employees, one of the trained individuals witnessed a cardiac arrest and performed life-saving CPR. In addition, *PulsePoint*-a system that alerts CPR trained individuals near a sudden cardiac arrest event and identifies locations of nearby AEDs (advocated by the EVMS service-learning team) was implemented in the Norfolk community.

The overall community impact of this service-learning initiative in a city of 250,000 is difficult to measure, but for the people who experience an out-of-hospital cardiac arrest in the presence of one of the 700 individuals trained in Bystander CPR, or for those who are assisted by individuals using *PulsePoint*, the chances of those individuals benefiting from lifesaving Bystander CPR have improved markedly. Meaningfully tying students’ limited service activity to population outcomes is a challenge that has not yet been completely surmounted, but one that is aggressively addressed with each initiative.

**Figure 4.**
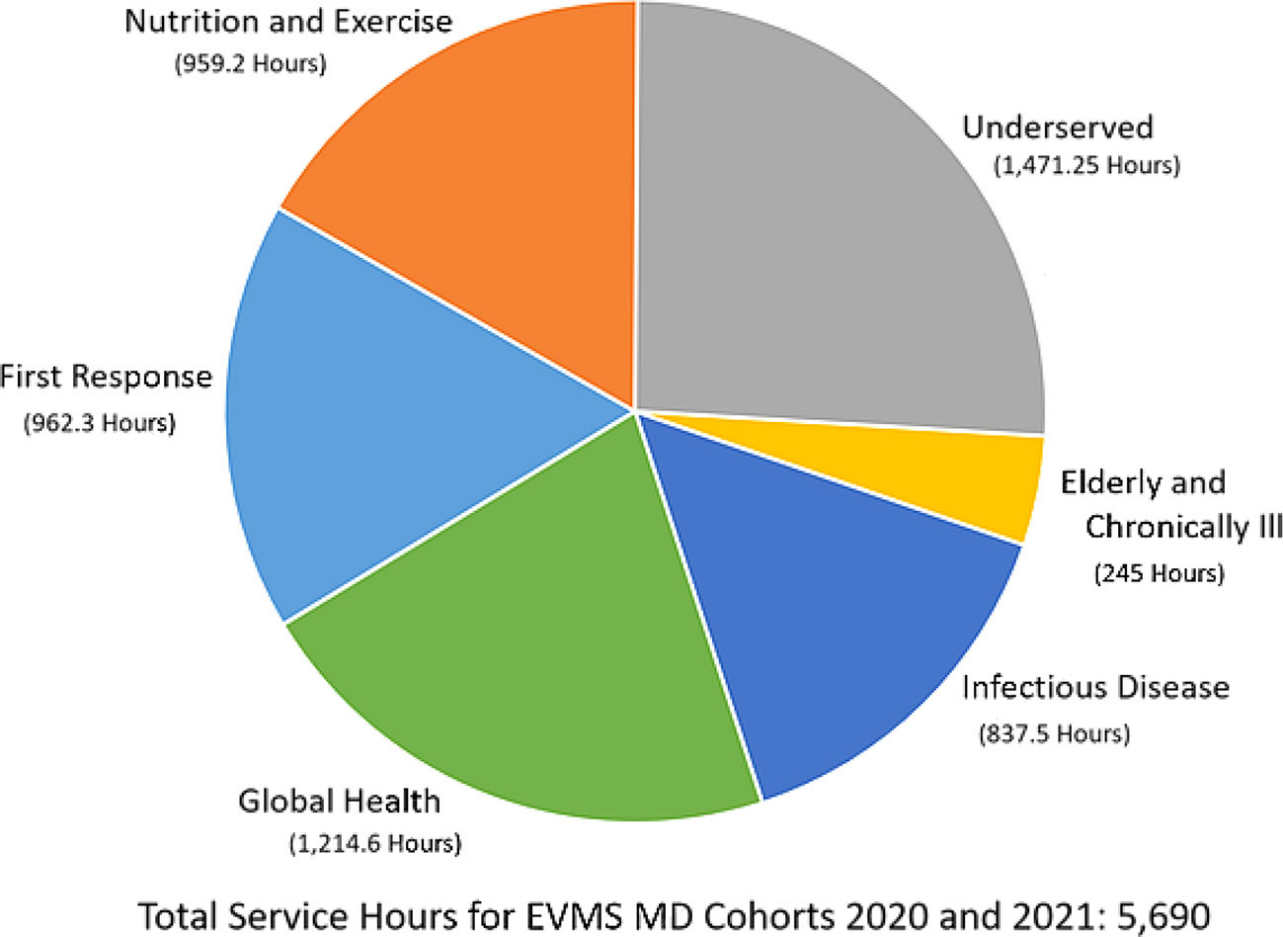
Distribution of EVMS student service hours across service pathways for school year 2017-2018. This graph includes the EVMS MD2020 and MD2021 cohorts.

**Service hours.** Service hours are a penultimate measure of impact, reflecting only student activity. The ultimate measures of impact are community health outcomes. But, service hours offer an idea of the magnitude of service. Observing the pace of service hours over the course of the school year can provide a valuable dashboard measure of initiative health. Two indicators that an initiative is healthy based on pace of activity are that a) observed activity (service hours) start to register early in the year, and b) the pace of student activity is distributed evenly throughout the year. Figure 4 presents the distribution of student service hours by service pathway.

### Student Evaluations

In late October and late April of the 2016-2017 academic year, and the 2017-2018 academic year, a seven-item Service-Learning course evaluation survey was administered to 274 students (α = .845). Participation in the survey was optional. The survey employed a five-point Likert-type response scale (5 = strongly agree, 1 = strongly disagree) for most items, and then asked three open-ended questions requiring narrative response. Descriptive and analytic data for Likert-type survey responses for one cohort of first year medical students is presented in Table 2, and Table 3 depicts that same data for the same cohort at the end of the second year of medical school. Consistent with principles articulated by Sullivan and Artino (2013) and Norman (2010), this analysis incorporates some statistical procedures once reserved exclusively for parametric data.

### Evaluation survey Likert-type items

• My Initiative’s orientation or initial training was very helpful. [Factor Name: Orientation]
• The expectation for my service time was clear. [Factor Name: Expectations]
• I have a voice in the future direction of my service initiative. [Factor Name: Voice]
• The mission of my service initiative is important to me. [Factor Name: Importance]
• I have a friend on my service-learning team. [Factor Name: Friend]
• My service initiative is interesting to me. [Factor Name: Interesting]
• I would recommend my service-learning initiative to new students. [Factor Name: Recommend]

**Table:**
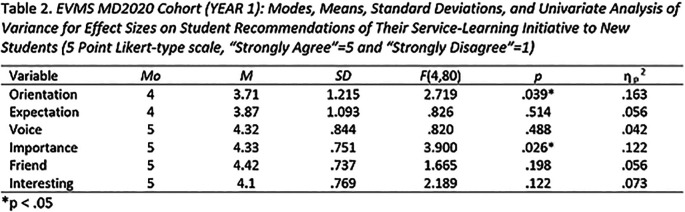


**Table:**
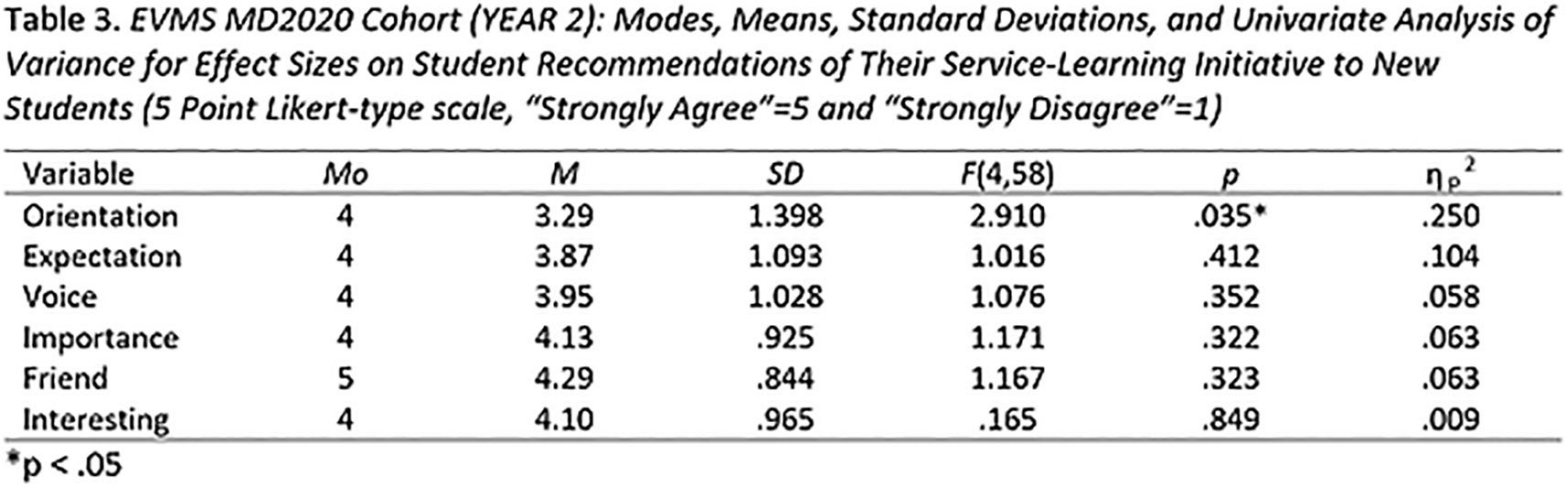


**Propensity for students to recommend their initiatives to new students.** The key measure of the quality of the student experience was whether or not the students would recommend their initiatives to new students. Main effects for “Orientation” were observed in both first and second year surveys. Orientation accounted for 16% of the variance for students recommending their initiative in the first year, and 25% of the variance for students recommending their initiative in second year. Taken together, these data support the contention that the quality of the initial student orientation to their initiative is a significant factor in students’ later recommending their initiatives to others.

A main effect for “Importance” observed in the first year survey accounted for 12% of the variance for students recommending their initiative.

**Figure 5.**
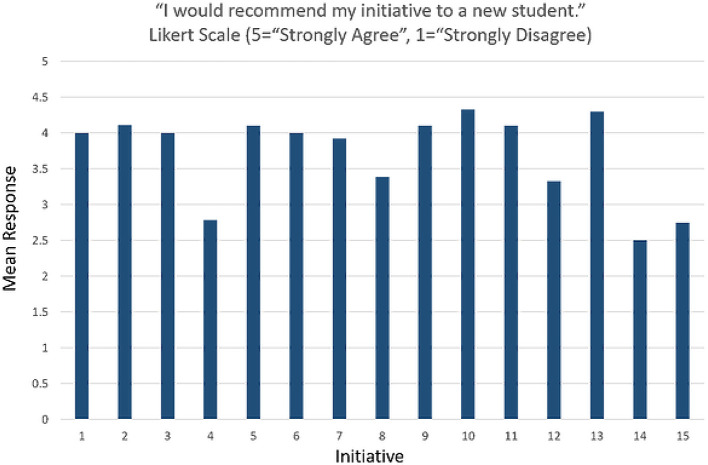
Mean student responses to the survey item “I would recommend my initiative to a new student.” Each numeral along the x-axis represents a unique service initiative. This graph includes data from both first and second year medical students (n=185).

On the end of year evaluation for 2017-2018 for first and second year medical students at EVMS, over 70% of students either “strongly agreed” or “agreed” with the item “I would recommend my service initiative to new students”, and another 6% responded with “disagree” or “strongly disagree” (n = 185). Figure 5 depicts student inclinations to recommend their initiative to new students according to initiative.

Note that in Figure 5 three of the 15 initiatives’ recommendation means were below 3.0, and two of the others were below 3.5. In all five cases, for various reasons, student activity within the initiatives started the year slowly. The initiatives with the highest means for students recommending the initiative were active from the beginning of the school year, oriented new students methodically, and boasted ongoing concrete service activities for students. They also led directly to moderately complex questions that were challenging for students: a perfect fit for Capstone research. The five initiatives with the lowest recommendation means were also low in perceptions that the orientation to the initiative was helpful.

That initiatives may go through an initial season (or transient season) of low student satisfaction does not necessarily mean the initiative should be discontinued or radically redesigned. There is a natural process of growth that mirrors that of innovation diffusion (see Rogers, 2003). For example, initiative number 14 was difficult to start, it was a complex environment in which medical students were to work with special needs children. The initiative took over half the academic year before students could actively engage, but the children with whom the students worked made significant gains in math and reading fluency over the last two months of the school year. Though the overall experience was frustrating for the medical students, significant progress was realized, and the way was prepared for them to do the same work from the beginning of the school year for the next year. The trials of the first year paved the way for a promising second year.

**Student Comments.** Student comments about their service as logged in the service-learning phone app, or in answer to an open-ended survey question, were generally positive, but negative feedback was received, as well. The following quotes provide a representation of the range of student comments.

**Table:**
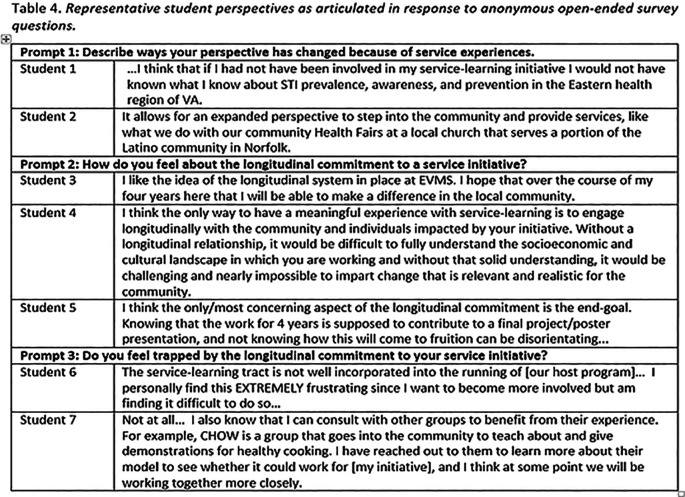


The comments provided in Table 4 are consistent with the survey feedback data synthesized in Tables 2 and 3. The majority of students (but not all) appear to buy into the longitudinal service element of this framework, and also the general pattern of service. But, a continued theme reflected in student comments is frustration with uncertainty. This uncertainty comes from three main sources: a) new initiatives come with a significant amount of uncertainty, and several students have participated in new initiatives, b) some uncertainty is still part of the overall program as it matures, and b) some of the uncertainty is connected with the unavoidable reality that if a requirement is open-ended-as Capstone is-there will be a realistic amount of uncertainty that is an expected part of the process.

## Discussion and conclusions

The sustained service framework is a four-year longitudinal framework for service-learning in undergraduate medical school. The opportunities afforded by this approach are numerous, including the opportunity for students to live the social determinants with real people in authentic contexts, to deepen expertise and credentials related to their service over time, to synthesize and consolidate their learning through a focused Capstone research experience, and to make a difference in the community’s priority need areas. At the same time, there are challenges associated with students associating with the same initiative over the course of medical school.

The approach starts with a careful community needs assessment, and structuring service opportunities to match priority community needs. Identifying service initiatives this way is likely a main reason 87% (80/92) of second year medical students and 81% (77/93) of first year medical students agreed or strongly agreed with the statement that their service mission is important on their end-of-year evaluation surveys.

The sustained service approach incorporates a motivation model that emphasizes student choice in selection of initiative, emphasizes intrinsic versus extrinsic motivation, and targets identity formation and paradigm development. The intentional inclusion of “escape routes” and optional activities decreases the sense that the service is a required curricular component, though it is.

Service-learning brings a certain amount of intrinsic ambiguity. Student perceptions about the helpfulness of the initial orientation appear to be the key variable in recommending the initiative to others. The strength of other variables affecting student perceptions of initiative quality appear to change over time. Survey data from early first year respondents indicates that the developing social relationships are important at that phase. The responses to the survey items “I have a friend on the team” and “I would recommend this initiative to others” were strongly correlated (r (123) = .56, p < .01). But, over the first two years of medical school, the importance of a strong orientation and the perceived importance of the mission become more salient.

**Practical implications.** These data lead to a practical conclusion regarding unique emphases during the different stages of service: in the initial stages, start from the beginning to articulate the vision and mission of an initiative, to lay out the path ahead. The initial orientation must provide new students with a vision for their service, and a sense for its importance. But, early in the service experience it is also important to emphasize opportunities for students to connect with each other and with the served population socially. As time progresses, insure the mission of the initiative is clear, practical, and advancing. In the end, students will evaluate the goodness of the initiative by progress against its mission goals.

**Future work.** There are several areas of fruitful exploration that accompany this framework. Three research questions that immediately arise and bear further study:

• How might Capstone best be structured to match both learner needs to build research skills but match AMA standards for scholarly research?
• Does the framework described here result in students choosing to engage in service later in their careers?
• To what degree are measurable improved community health outcomes a realistic target for service-learning and how might those gains best be articulated?
• How important is a strong orientation to student desires to recommend the initiative to new students? How do students evaluate an orientation as ‘strong’?

**Conclusion.** Fredrick Buechner (2017) asserted that our personal mission is “the place where [our] deep gladness and the world’s deep hunger meet.” For medical students, the sustained service framework provides an excellent medium for sounding the depths of both their professional desires and passions in practical settings, as well as exploring local expressions of the world’s great need. It is a perfect venue for exploring personal mission, the social determinants of health, and useful community-oriented skills and knowledge.

This project used data from routine program evaluations and therefore was exempt from institutional ethics approval.

## Take Home Messages

• Sustained service presents unique opportunities for expertise development, identity formation, and personal relationship.
• Service need not be “random acts of kindness,” there is value in using a rigorous community engagement process to hear the voices of community members and leaders as priorities are developed, and then using objective sources of data (e.g., Community Health Assessments and other data-rich reports) to guide conclusions. We call this approach “Community-Driven, Data Supported” in the sustained service framework.
• Students need a clear orientation to their service, it is a key variable they will use in later evaluating whether or not they would recommend the service to others.
• Student response to sustained service has been positive, but it isn’t everyone’s preference.
• Systematically planning identity-forming elements may help establish a community-responsive physician identity. These inputs can be subtle and easy to implement, like including visual insignia for groups, or providing opportunities for success in the context of community service.
• Role models play a key role in identify formation, so great care should be taken in recruitment of initiative facilitators.
• Incorporating a synthesizing Capstone experience is a valuable way to cap the four year longitudinal service-learning experience. Have students focus on the population they served.

## Notes On Contributors

**Don Robison, Ph.D., CPT** is the Director of Service-Learning at Eastern Virginia Medical School.

**Alexandra Leader, MD, MPH** is the Director of Global Health at Eastern Virginia Medical School.

**Maryanne Gathambo, MPH** is the Associate Director of Service-Learning at Eastern Virginia Medical School.

**Erin Madison** is a member of the Eastern Virginia Medical School MD Class of 2021.

**Alicia St. Thomas, MA** is a member of the Eastern Virginia Medical School MD Class of 2020.
