# Supplementary material for: Sustained service: a community-driven framework for longitudinal service-learning
Source: MedEdPublish (2016). 2018 Jun 5;7:112. [Version 1] doi: 10.15694/mep.2018.0000112.1 (PMC10699387; doi:10.15694/mep.2018.0000112.1)
Supplement: Peer review history

# Reviewer report: Reviewer response for version 1

**DOI:** 10.21956/mep.19585.r29072

**Published:** 2019-03-14

**Authors:** Richard Hays

**Affiliations:** 1 James Cook University

This review has been migrated. The reviewer awarded 5 stars out of 5 I enjoyed this paper. It is affirming to read that student projects can make a difference, particularly if they are matched to community needs and are sustained over a reasonable time period. The model here is based on individual students participating for all 4 years of their medical program, but other models ‘tag-team’ students over long periods of time, with individual students partipating for shorter periods, picking up where others stop and then handing over to others when their engagement finishes. Many community development projects take years to mature and have impact, yet students can gain from participation at any stage where they feel a sense of ownership. It would be interesting to explore the career outcomes of participating students: are they different to students in other programs?

Reviewer Expertise:

NA

No decision status is available

# Reviewer report: Reviewer response for version 1

**DOI:** 10.21956/mep.19585.r29073

**Published:** 2018-06-07

**Authors:** P Ravi Shankar

**Affiliations:** 1 American International Medical University

This review has been migrated. The reviewer awarded 5 stars out of 5 I enjoyed thoroughly this detailed description of sustained service initiatives and learning through service at the Eastern Virginia Medical School in the United States. The initiative is designed to help with student learning and at the same time improve community health outcomes. The use of the pathways and learning/service initiatives has been well described. The process of talking to community leaders and community members before using objective data sources seems logical. Use of a phone app for students to report and for the leaders to monitor their service seems to be an effective solution for a requirement which at times could be complex. Role-models as mentioned by the authors are extremely important. The four year duration provides students with the opportunity to be ‘immersed’ in the community and develop strong connections. In Nepal, students do a two or three week community diagnosis project where they identify the health needs of a rural or semi-rural community. They revisit the community periodically during their course of study. Sustained service shares some similarities with the community diagnosis projects but also has differences. The greater use of technology and relatively easier access to resources is one important one. I congratulate the authors on this program and look forward to future developments. All medical educators will find this project and the description to be of interest.

Reviewer Expertise:

NA

No decision status is available
